# Decoding monocyte heterogeneity in sepsis: a single-cell apoptotic signature for immune stratification and guiding precision therapy

**DOI:** 10.3389/fphar.2025.1675887

**Published:** 2025-10-03

**Authors:** Wenjuan Duan, Qi Chen, Wei Li, Haiying Zhou, Xiaorong Deng, Yan Zhang

**Affiliations:** ^1^ Department of Pulmonary and Critical Care Medicine, First Affiliated Hospital of Chengdu Medical College, Chengdu, Sichuan, China; ^2^ School of Clinical Medicine, Chengdu Medical College, Chengdu, Sichuan, China; ^3^ Department of Neurology, First Affiliated Hospital of Chengdu Medical College, Chengdu, Sichuan, China; ^4^ Department of Gastroenterology, First Affiliated Hospital of Chengdu Medical College, Chengdu, Sichuan, China; ^5^ Department of Rehabilitation Medicine, First Affiliated Hospital of Chengdu Medical College, Chengdu, Sichuan, China; ^6^ Department of Pediatrics, First Affiliated Hospital of Chengdu Medical College, Chengdu, Sichuan, China

**Keywords:** sepsis, monocyte, classical monocytes, ScRNA-seq, bulk RNA-seq, apoptotic

## Abstract

**Background:**

The effectiveness of immunomodulatory therapies in sepsis is often hampered by profound and patient-specific immune heterogeneity. Classical monocytes play a central role in the progression toward sepsis-induced immunoparalysis, with their apoptotic rate serving as a sensitive marker of immune dysfunction. Traditional bulk transcriptomic approaches fail to resolve this complexity. Here, we harness single-cell RNA sequencing to delineate the apoptotic landscape of classical monocytes and identify robust molecular biomarkers for immunological stratification and targeted intervention.

**Methods:**

We integrated single-cell and bulk transcriptomic data from four independent cohorts. A machine learning pipeline incorporating SVM, RF, XGB, and GLM algorithms was used to identify hub genes associated with monocyte apoptosis. A diagnostic nomogram was constructed based on the selected gene signature and validated across external datasets. Clinical relevance was confirmed through Western blot analysis of purified monocytes from sepsis patients and healthy controls.

**Results:**

A four-gene signature (G0S2, GZMA, ITM2A, PAG1) emerged as a specific apoptotic fingerprint of classical monocytes. The diagnostic model based on these signature genes demonstrated excellent discriminatory performance, effectively stratifying patients into high-risk and low-risk groups (AUC >0.8 across multiple validation cohorts), with each risk group exhibiting distinctly different immune states. High-risk patients exhibited a pro-inflammatory transcriptomic profile with elevated apoptotic pathway activity (e.g., neutrophil degranulation), whereas the low-risk group showed enrichment in adaptive immunity and T cell receptor signaling. Protein-level validation in clinical samples corroborated the transcriptomic findings.

**Conclusion:**

This study elucidates a critical facet of immune heterogeneity in sepsis through the identification of a validated, four-gene apoptotic signature in classical monocytes. Beyond its diagnostic utility, this signature serves as a molecular indicator of immune state, enabling refined patient stratification. These findings lay the groundwork for precision immunopharmacology, where apoptosis-targeted or anti-inflammatory therapies can be tailored to individual immune profiles.

## 1 Introduction

Sepsis represents a critical global public health challenge. According to statistics, there were approximately 48.9 million cases of sepsis worldwide in 2017, with a mortality rate of 22.5%, accounting for nearly 20% of all global deaths (Rudd et al., 2020; Gavelli et al., 2021). The elderly, individuals with compromised immune function, and patients with underlying conditions such as diabetes, malignancies, or chronic kidney disease are particularly at high risk for developing sepsis (Meyer and Prescott, 2024). Although advances in the treatment and diagnosis of sepsis have been achieved in high income countries in recent years, the incidence of sepsis continues to rise (Fleischmann et al., 2016). Its persistently high incidence, elevated mortality, frequent complications, and poor prognosis remain significant concerns. Therefore, identifying novel and sensitive biomarkers for early diagnosis and timely intervention is essential for improving patient outcomes.

While sepsis involves a systemic immune response engaging multiple cell lineages, classical monocytes are uniquely positioned at the nexus of hyperinflammation and subsequent immunoparalysis. Their functional state serves as a critical indicator of overall immune competence, and their apoptotic rate is a sensitive marker of the immune dysregulation that drives sepsis progression (Giamarellos-Bourboulis et al., 2020). Therefore, focusing on this specific cell type offers a granular yet clinically relevant window into the pathophysiological mechanisms of sepsis, providing a strategic advantage for identifying potent biomarkers and therapeutic targets.

In the complex pathophysiological process of sepsis, monocytes serve not only as key regulators of the innate immune response but also play a central role throughout the progression of sepsis-induced immunosuppression. Acting as primary components of innate immune surveillance, the functional status of monocytes is a critical indicator of overall immune competence. In the early stages of sepsis, following a severe infectious insult, the host immune response rapidly shifts from an initial hyperinflammatory phase to a state of immunosuppression—a transition that is pivotal in driving disease progression and secondary infections. A hallmark of this transition is “monocyte reprogramming” (Yao et al., 2023): under persistent stimulation by pathogen-derived products such as endotoxins, production of pro-inflammatory cytokines (e.g., TNF-α, IL-6) by monocytes is significantly suppressed, while the secretion of anti-inflammatory factors such as IL-10 is markedly promoted. Immunosuppression is largely facilitated by the phenotypic shift of monocytes toward an anti-inflammatory state (Liu et al., 2019). Concurrently, the downregulation of human leukocyte antigen-DR (mHLA-DR) expression on the surface of monocytes represents another key feature of this suppressed immune state (Washburn et al., 2019). mHLA-DR expression directly reflects monocyte activation, and its reduction is widely recognized as a biomarker of immunosuppression, correlating strongly with increased mortality and infection risk in patients with sepsis (Monneret et al., 2006; Venet and Monneret, 2018; Ożańska et al., 2020). Recent studies further suggest that the severity of this immunosuppressive state directly influences the therapeutic efficacy of immune adjuvants (e.g., granulocyte-macrophage colony-stimulating factor, GM-CSF) (Leijte et al., 2020; Bodinier et al., 2021).

In addition to functional impairment, the depletion of immune cell populations—particularly monocyte apoptosis—is another major driver of immunosuppression in sepsis (Nedeva et al., 2019). Studies have demonstrated that apoptosis of peripheral blood monocytes is significantly elevated in patients with sepsis (Reséndiz-Martínez et al., 2017), leading to a marked reduction in their numbers. This quantitative loss directly compromises key immune functions, including antigen presentation, cytokine secretion, and pathogen phagocytosis, thereby rendering the host more susceptible to secondary infections and perpetuating a vicious cycle of immunosuppression (Mohri et al., 2006). Importantly, both monocyte dysfunction and apoptosis serve not only as critical biomarkers for prognostic assessment but also as promising therapeutic targets. Current research has focused on restoring monocyte function and delaying apoptosis through the use of immunostimulatory agents—such as granulocyte-macrophage colony-stimulating factor (GM-CSF) and interferon-gamma (IFN-γ)—or apoptosis inhibitors. These strategies have shown potential in improving clinical outcomes in septic patients (Meisel et al., 2009). Therefore, a deeper understanding of monocyte apoptosis and the development of targeted interventions may offer novel and effective immunomodulatory approaches for the treatment of sepsis.

Early recognition and timely intervention are critical for improving the survival of patients with sepsis. Compared to conventional inflammatory biomarkers as exemplified by C-reactive protein (CRP) and procalcitonin (PCT), the level of monocyte apoptosis offers a more direct and sensitive reflection of the immune dysregulation characteristic of sepsis. scRNA-seq represents a cutting-edge approach that enables the clustering of cells to investigate intergroup differences in gene expression and cellular progression (Davis et al., 2021). This work combined single-cell and bulk RNA sequencing data and employed multiple machine learning algorithms to systematically identify key genes associated with monocyte apoptosis in sepsis. Utilizing these genes, we developed and validated a diagnostic model for predicting sepsis risk. Further analyses revealed significant differences in immune infiltration patterns and microenvironment features between patient groups stratified by risk. Notably, this study is the first to incorporate monocyte apoptosis into the early diagnostic framework for sepsis, representing a paradigm shift from the traditional focus on inflammatory responses to a deeper investigation of immune dysfunction. This novel approach provides new insights for achieving earlier and more precise diagnosis and risk stratification, offering the potential to gain valuable time for clinical intervention and improve patient outcomes.

## 2 Materials and methods

### 2.1 Download and processing of conventional transcriptome data

For the purpose of model training, the GSE65682 dataset was sourced from the GEO repository and applied as the primary training cohort in this study. This dataset includes 430 sepsis samples and 42 healthy control samples. The GSE26440, GSE95233, and GSE26378 datasets were used as independent validation cohorts, comprising 98 sepsis samples and 32 healthy controls, 102 sepsis samples and 22 healthy controls, and 82 sepsis samples and 21 healthy controls, respectively. During data preprocessing, samples with missing gene expression values in more than 50% of genes were excluded. Additionally, genes with low expression frequency—defined as presence in less than half of the retained samples—were filtered out to enhance the robustness of the analysis.

### 2.2 Acquisition and preprocessing of single-cell RNA-Seq data

GSE167363, a single-cell transcriptomic dataset, was downloaded from the GEO database to support downstream analyses. This dataset comprises 12 sepsis samples and 2 healthy control samples. The “Seurat” R package (version 5.1.0) was utilized for single-cell data analysis, with normalization conducted via the “NormalizeData” function. Mean expression and dispersion parameters were used to screen for highly variable genes. Cell clustering was carried out using the “FindClusters” algorithm, which is optimized based on the shared nearest neighbor (SNN) modularity approach, across 30 principal components (PCs), with the resolution parameter set to 1.0, resulting in 21 distinct clusters. Subsequently, t-distributed stochastic neighbor embedding (t-SNE) was applied using the “RunTSNE” function to visualize cellular distributions. Differentially expressed genes (DEGs) were identified with the “FindAllMarkers” function in Seurat, enabling cluster annotation and evaluation of cell-type proportions. After PCA and clustering were conducted on the normalized expression data, Uniform Manifold Approximation and Projection (UMAP) was used for dimensionality reduction visualization. The number of principal components used was determined according to the elbow curve, and clustering was conducted at multiple resolutions. Automatic annotation of cell clusters was performed using both SingleR and ScType, allowing for robust identification and cross-validation of cell types. The final cell-type annotations were integrated and used for downstream analyses and visualizations.

### 2.3 Analysis of AUC score of apoptosis related genes

A total of 21,896 apoptosis-related genes were retrieved from the GeneCards database (https://www.genecards.org/). Gene set enrichment analysis (GSEA) was performed using the AUCell package to evaluate the expression activity of apoptosis-related genes at the single-cell level. Specifically, enrichment of apoptosis-associated genes was quantified per cell by calculating the corresponding area under the curve (AUC). Cells with higher AUC scores were considered to exhibit elevated expression of apoptosis-associated genes.

### 2.4 Cell communication analysis

To explore cell-to-cell signaling networks, we utilized the CellChat R package (version 1.6.1), with a particular focus on interactions between classical monocytes and other immune cell populations. CellChat infers cell–cell communication networks by quantifying interactions between receptor–ligand binding events and their downstream signaling pathways. The results were visualized using heatmaps, which illustrated the enrichment and intensity of ligand–receptor interactions across different cell types. In addition, using CellChat, we quantified the relative impact of endogenous and exogenous signaling routes within each cell type, thereby revealing differential cellular responses to external cues within the sepsis-associated microenvironment.

### 2.5 Time series analysis is proposed

We performed pseudotime analysis using the Monocle2 toolkit, focusing on genes exhibiting elevated expression variability (dispersion ≥1) and average expression levels ≥0.1. This analysis enabled the construction of pseudotemporal trajectories, outlining the developmental progression of peripheral blood cells in sepsis and capturing dynamic changes in cellular states. Branch expression analysis modeling was further applied to explore gene expression alterations involved in cell fate decisions. A heatmap was generated to visualize the expression patterns of the top 10 apoptosis-related genes across different trajectory branches, highlighting lineage-specific transcriptional changes. Notably, we conducted an independent pseudotime trajectory analysis specifically for classical monocytes, which revealed their developmental progression and differentiation within the sepsis-associated microenvironment.

### 2.6 Screening of DEGs

To identify cell type–specific marker genes, differential expression analysis was performed using the FindAllMarkers function in the Seurat package (version 5.1.0). The parameters were set as follows: min.pct to 0.1 and logfc.threshold to 0.25. This threshold corresponds to a natural log fold change, which is the default for the function. For final screening and consistency with bulk RNA-seq analysis, we retained genes with an adjusted P-value <0.05 and an absolute log_2_ fold change ≥0.58.

Differential expression analysis for bulk transcriptomic data was carried out via the limma R package (version 3.58.1). To address multiple comparisons, adjusted P values were calculated using the Benjamini–Hochberg procedure. Genes meeting the criteria of FDR <0.05 and an absolute log_2_ fold change ≥0.58 were retained for further analysis. DEGs common to the GSE65682, GSE26440, GSE95233, and GSE26378 datasets were identified by intersecting DEG lists, resulting in a consistent set of differentially expressed genes between septic and normal blood samples obtained from GEO.

### 2.7 Screening of genes related to classical mononuclear cell apoptosis in sepsis

We applied Venn analysis to extract common genes shared across three gene sets, one of which consisted of DEGs distinguishing sepsis from healthy controls in GEO datasets, classical monocyte-associated genes, and apoptosis-related genes. The overlapping genes were defined as apoptosis-related genes within classical monocytes collected from blood samples of patients suffering from sepsis (referred to as inter genes).

### 2.8 Establishment and verification of diagnostic risk model

To uncover diagnostic biomarkers relevant to sepsis, four machine learning algorithms were employed using a combination of R packages, including caret, DALEX, e1071, randomForest, and glmnet. Four widely adopted machine learning algorithms—SVM, RF, XGB, and GLM—were employed to construct the diagnostic model. The top 15 genes ranked by importance in each algorithm were extracted, and the intersecting genes among all four methods were defined as hub genes for diagnostic model construction.

Machine learning models were developed employing the caret R package (version 6.0.91), incorporating RF, SVM, GLM, and XGB algorithms. RF operates by building numerous uncorrelated decision trees and aggregating their outputs for classification or regression. SVM constructs a hyperplane in the feature space to optimally separate negative and positive samples based on the maximum margin principle. GLM extends traditional linear regression to allow flexible modeling of relationships between the outcome variable and both categorical and continuous predictors. XGBoost, a powerful gradient boosting framework, constructs decision trees sequentially to minimize classification error and avoid overfitting. Each model was constructed using default parameters and validated via five-fold cross-validation. Interpretability analysis was conducted using DALEX (version 2.4.0), providing graphical visualizations of prediction distributions and feature importance across the four algorithms.

Univariate and multivariate logistic regression analyses were conducted using the broom package (version 1.0.6). A diagnostic RiskScore was calculated based on the regression coefficients using the formula: RiskScore = β_1_X_1_ + β_2_X_2_ + … + β_n_X_n_, where β represents the regression coefficient and X denotes gene expression values. RiskScore calculations were applied to stratify subjects from both training and validation cohorts into distinct risk categories. The model’s diagnostic capability was evaluated through receiver operating characteristic (ROC) curves, which reflect its overall predictive performance.

### 2.9 Construction of the nomogram model

Based on the expression patterns of the identified hub genes, we carried out both univariate and multivariate logistic regression analyses to screen for independent predictors associated with sepsis. These selected variables were then incorporated into the construction of a diagnostic nomogram. Model building and visualization were conducted using the rms package (v6.7-1). The resulting nomogram was developed to estimate the probability of sepsis occurrence. To evaluate the agreement between predicted and actual outcomes, a calibration plot was generated. The decision curve analysis (DCA) was applied to assess the clinical utility of the model across various threshold levels. Additionally, a clinical impact curve was plotted to further validate the model’s effectiveness in identifying individuals at high risk. Finally, the nomogram’s discriminative capacity was tested in three external validation datasets—GSE26440, GSE95233, and GSE26378—using ROC curve analysis.

### 2.10 Immune microenvironment analysis

We employed the CIBERSORT and single-sample GSEA (ssGSEA) algorithms to evaluate differences in immune cell infiltration between high and low-risk groups, and among subgroups with high expression levels of the 4 hub genes.

### 2.11 GSEA analyse

GSEA was performed to explore the enrichment from the curated gene set database c2.all.v2024.1.Hs.symbols between high- and low-RiskScore groups. Enrichment results were considered significant when the |NES| exceeded 1 and P value was <0.05, ensuring the robustness and relevance of the enrichment results.

### 2.12 Clinical sample collection and mononuclear cell separation

The Ethics Committee of the First Affiliated Hospital of Chengdu Medical College approved the study protocol, and written informed consent was obtained from all enrolled individuals. Six patients diagnosed with sepsis and admitted to our hospital between February and April 2025 were enrolled as the experimental group. Six age- and sex-matched healthy volunteers recruited during the same period served as the control group. For each subject, a 10 mL sample of fasting peripheral venous blood was obtained from each participant in the early morning. Ficoll density gradient centrifugation was employed to isolate peripheral blood mononuclear cells (PBMCs). Monocytes were subsequently purified using CD14 magnetic bead–based selection. Flow cytometry confirmed that the purity of the isolated monocytes exceeded 95% in all samples.

### 2.13 Western blot assay

Total protein was extracted from isolated monocytes, and protein concentration was determined using the bicinchoninic acid (BCA) assay. Following separation by SDS-PAGE, proteins were transferred onto polyvinylidene difluoride (PVDF) membranes. The PVDF membranes were incubated overnight at 4 °C with primary antibodies targeting G0S2 (Abcam, ab236113; 1:1000), GZMA (Proteintech, 11288-1-AP; 1:1000), ITM2A (Proteintech, 14407-1-AP; 1:1000), and PAG1 (Proteintech, 25029-1-AP; 1:1000). After washing, membranes were treated the next day with horseradish peroxidase (HRP)-conjugated secondary antibodies, followed by signal detection using an enhanced chemiluminescence (ECL) system. Densitometric analysis of protein bands was performed using ImageJ software. The expression levels of target proteins were normalized to β-actin, which served as the internal loading control.

### 2.14 Statistical analysis

All statistical analyses were carried out using R software. Group comparisons between two datasets were evaluated using the Wilcoxon rank-sum test, while differences among three or more groups were assessed via the Kruskal–Wallis test. Logistic regression and survival analyses were performed, with survival differences evaluated using the log-rank test. Statistical significance in graphical outputs was denoted as follows: ns (not significant, p ≥ 0.05), * (p < 0.05), ** (p < 0.01), and *** (p < 0.001).

## 3 Results

### 3.1 Single-cell transcriptomic analysis

#### 3.1.1 Dimensionality reduction, clustering, and annotation of single cells

**FIGURE 1:**
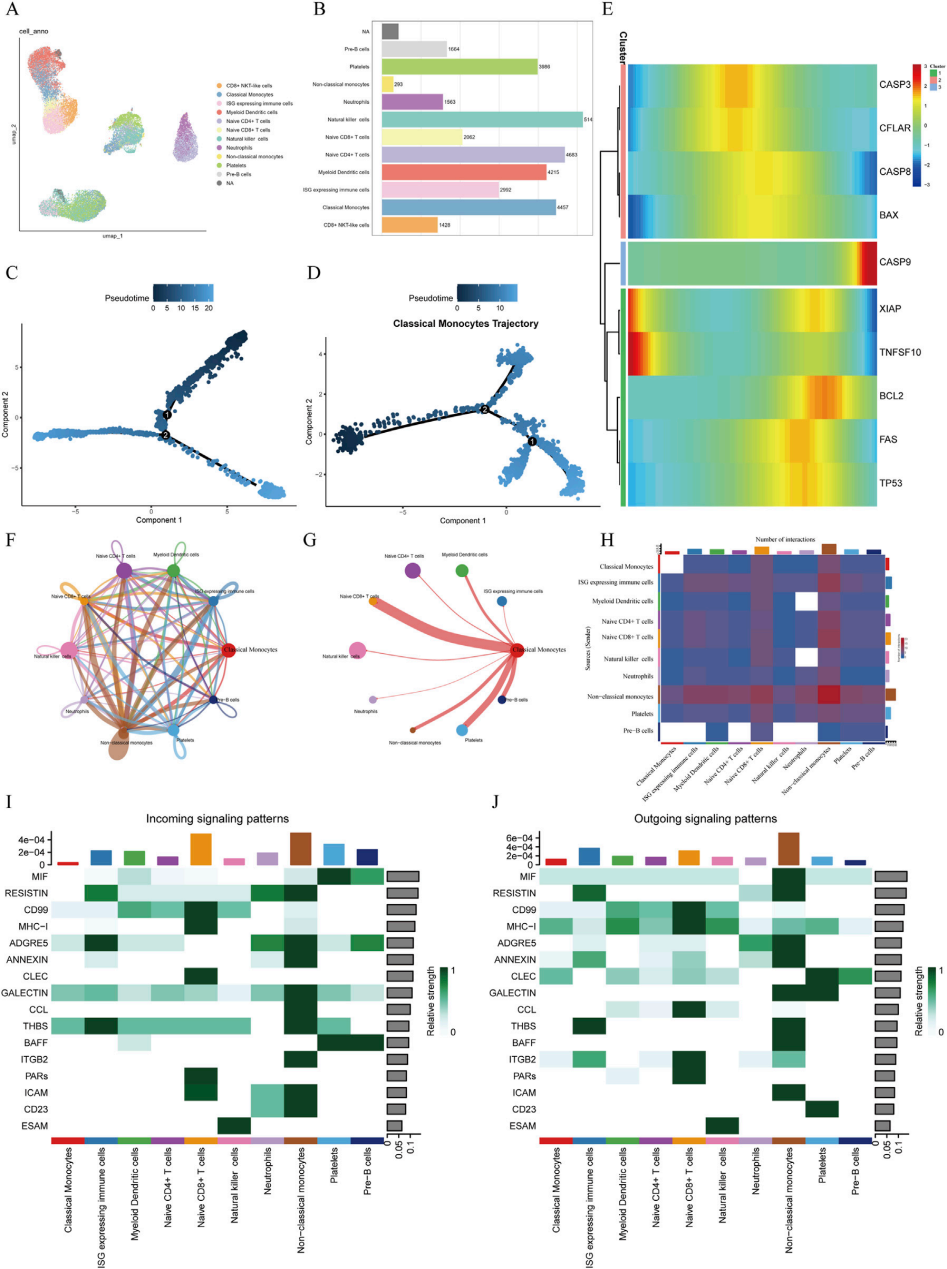
Single-cell transcriptomic analysis of sepsis **(A)** UMAP visualization depicting cell clusters identified via cell-specific markers, revealing 12 distinct cell types including classical monocytes, non-classical monocytes, and natural killer cells. **(B)** Bar graph illustrating the distribution of cell subsets in sepsis, highlighting Naive CD4^+^ T cells, Natural killer cells, and Classical monocytes as the most abundant populations. **(C)** Pseudotime trajectory analysis showing cellular developmental trajectories and state transitions. **(D)** Pseudotime trajectory of classical monocytes, depicting their differentiation process within the septic microenvironment. **(E)** Temporal expression profiles of apoptosis-related genes across pseudotime. **(F)** Cell-cell communication network demonstrating interactions among various cell types. **(G)** Interaction diagram highlighting communication patterns between classical monocytes and other immune cells. **(H)** Heatmap illustrating the intensity and patterns of intercellular communication among different cell subsets. **(I,J)** Circle plots illustrating the incoming **(I)** and outgoing **(J)** signaling patterns for classical monocytes. The plots show the relative contribution of other cell types in sending signals to (incoming) and receiving signals from (outgoing) classical monocytes across different signaling pathways.

To characterize the transcriptomic profiles of distinct immune cell subsets in sepsis, we analyzed the GSE167363 single-cell RNA-seq profile. t-SNE analysis partitioned the cells into 21 distinct clusters. According to the expression of known cell-specific markers, these clusters were annotated, resulting in the identification of 12 distinct immune cell types. Among them, classical monocytes accounted for 13.6% (n = 4,457) of the total cells, while non-classical monocytes represented only 0.9% (n = 293). UMAP visualization demonstrated well-defined clustering and clear separation between cell populations, indicating high accuracy and reliability of the annotation (Figure 1A). In addition, a bar plot of cell counts further illustrated the distribution of each cell subset across the samples (Figure 1B). Notably, such as CD4^+^ naïve T cells and natural killer (NK) cell populations, and classical monocytes were markedly enriched in the sepsis condition.

#### 3.1.2 Pseudotime analysis

To infer the developmental continuum of classical monocytes in the septic microenvironment, we performed pseudotime analysis. The resulting trajectory map illustrates a potential progression of cellular states rather than a direct timeline of the clinical disease course (Figure 1C). The analysis revealed that classical monocytes were distributed along the principal developmental branch, which we conceptually divided into early, middle, and late pseudotime stages. This distribution suggests that these cells undergo significant transcriptomic shifts that may correlate with different phases of the host response during sepsis (Figure 1D). To further elucidate the temporal dynamics of apoptosis-related genes, ten such genes were mapped along the pseudotime trajectory. Clustering analysis revealed three distinct expression patterns: Pattern 1 (CASP3, CFLAR, BAX): high expression in the early stage followed by a gradual decline; Pattern 2 (CASP9): stable expression during the early and middle stages with a marked increase in the late stage; Pattern 3 (XIAP, TNFSF10, BCL2, FAS, TP53): low expression in the early stage, peaking in the middle stage, and decreasing in the late stage (Figure 1E).

#### 3.1.3 Cell–cell communication analysis

The results of cell–cell communication analysis revealed a complex network of ligand–receptor interactions among various immune cell populations within the sepsis-associated immune microenvironment (Figure 1F). Overall, non-classical monocytes and neutrophils exhibited prominent signaling activity as major signal senders, engaging in high-frequency interactions with nearly all other immune cell types. This suggests their central regulatory role in the inflammatory network during sepsis. Notably, the number of outgoing signals from non-classical monocytes to neutrophils peaked, indicating a potential signal amplification role in the acute inflammatory response.

In contrast, classical monocytes displayed a “low-sender, high-receiver” communication profile. Although they participated in relatively fewer outgoing interactions, the strength of incoming signals was markedly elevated, particularly from non-classical monocytes and ISG high cell populations (Figures 1G,H). Ligand–receptor pathway analysis further indicated that classical monocytes primarily received signals through pathways such as GALECTIN and THBS, which are associated with apoptosis, adhesion, and chemotaxis. These exogenous signals may contribute to the induction of programmed cell death or functional exhaustion of classical monocytes during the course of sepsis (Figures 1I,J).

### 3.2 Identification of apoptosis-related genes in classical monocytes associated with sepsis

**FIGURE 2:**
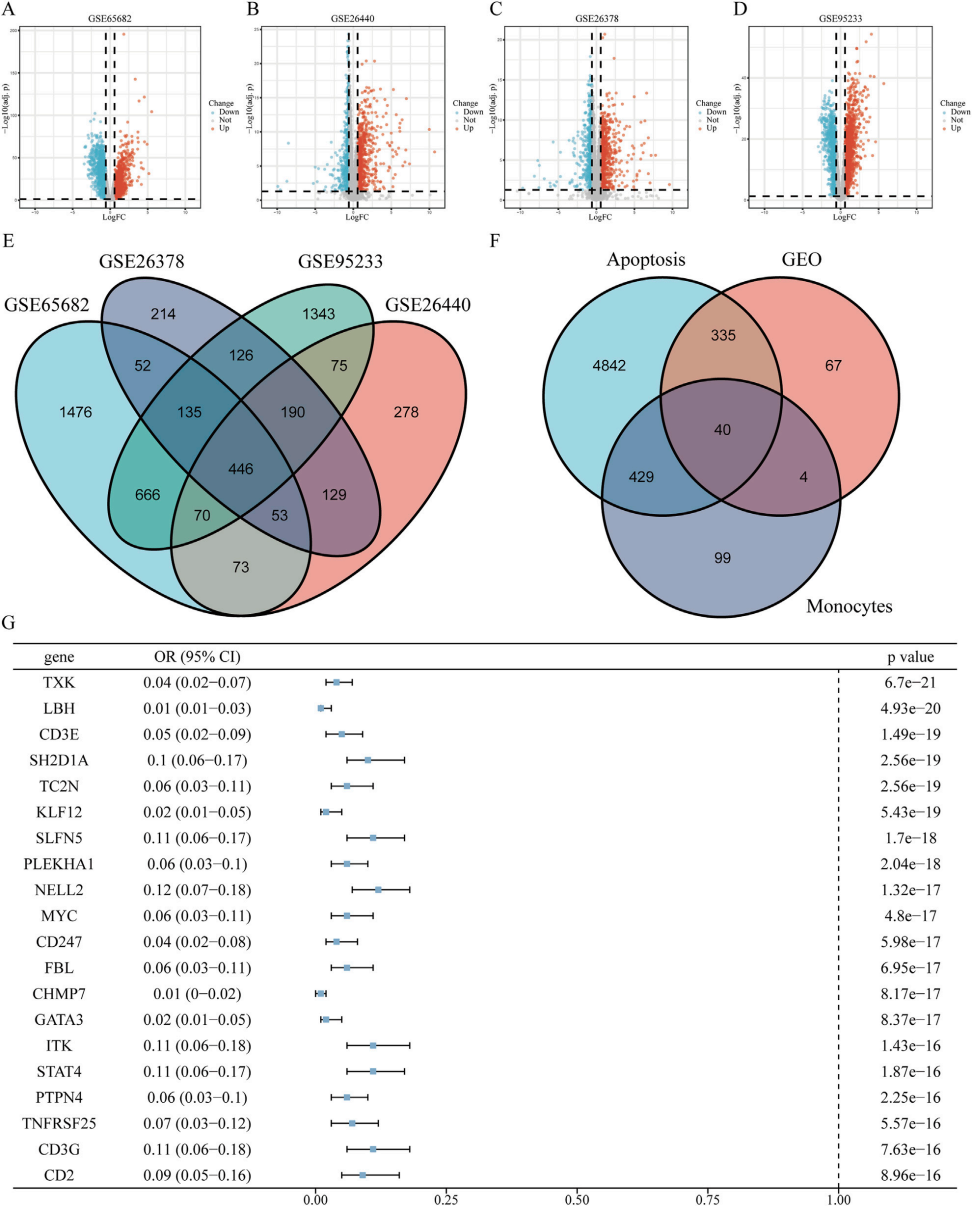
Screening of apoptosis-related genes in classical monocytes during sepsis **(A–D)** Volcano plots displaying differentially expressed genes (DEGs) from datasets GSE65682, GSE26440, GSE95233, and GSE26378, highlighting up- and downregulated genes. **(E)** Venn diagram illustrating the intersection of DEGs identified across the four datasets. **(F)** Venn diagram showing overlaps among classical monocyte-specific genes, apoptosis-related genes, and identified DEGs. **(G)** Forest plot of univariate logistic regression analysis for the top 20 significant apoptosis-related genes expressed in classical monocytes (ranked by P-value). The complete forest plot for all 40 genes is available in Supplementary Figure S1.

By identifying DEGs between healthy individuals and patients with sepsis, we intersected DEGs from four GEO datasets—GSE65682, GSE26440, GSE95233, and GSE26378—and obtained a total of 446 shared DEGs (Figures 2A–E). Further single-cell differential expression analysis of sepsis samples yielded 572 classical monocyte–associated genes. We extracted 21,896 genes related to apoptosis from the GeneCards resource. Intersecting these three gene sets resulted in the identification of 40 apoptosis-related genes specifically expressed in classical monocytes in sepsis (Figure 2F). Univariate logistic regression analysis of these 40 intersecting genes revealed indicating that 3 acted as risk contributors, whereas 37 served protective roles (Figure 2G).

### 3.3 Selection of hub genes involved in apoptosis of classical monocytes in sepsis

**FIGURE 3:**
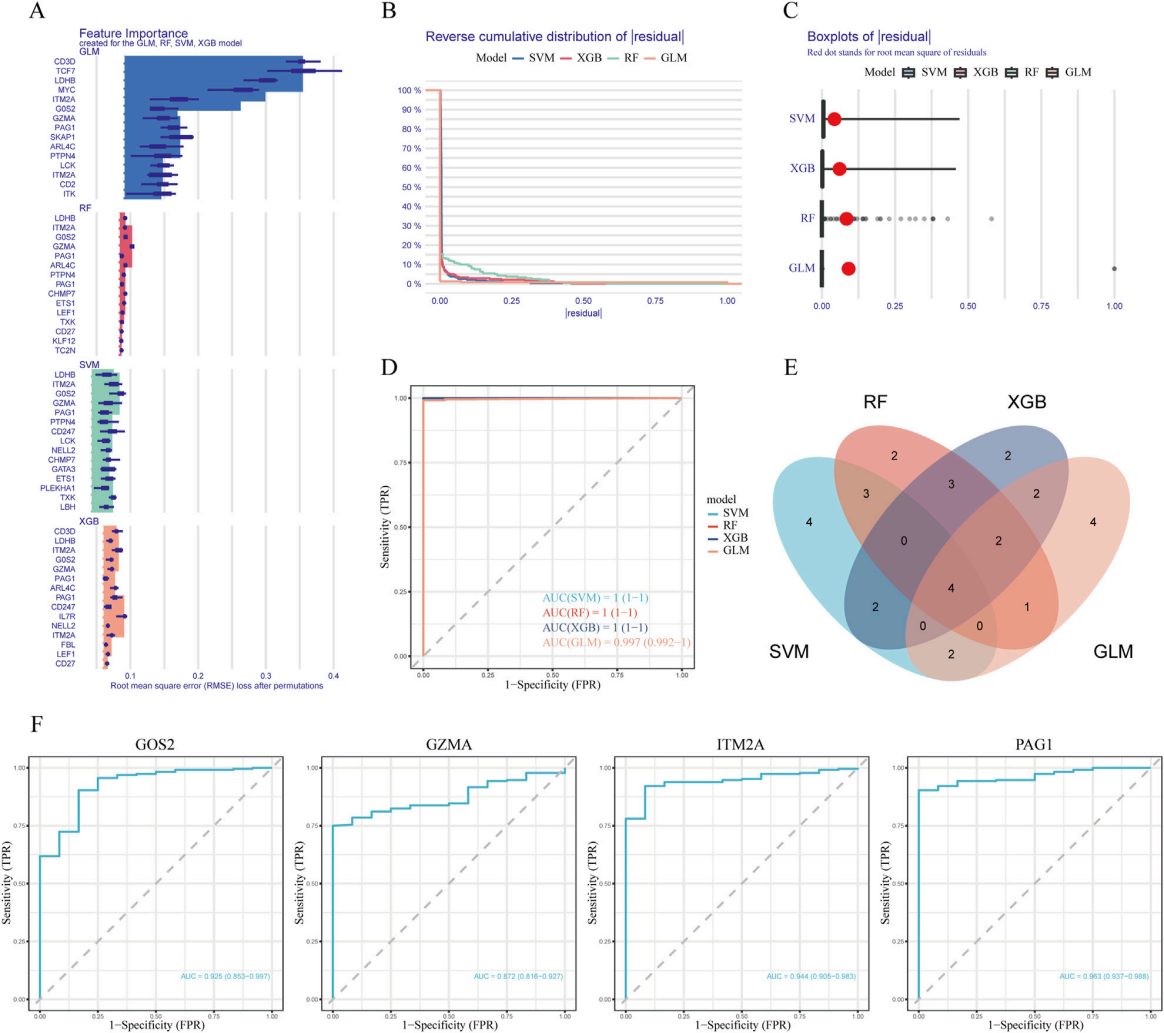
Machine learning modeling and hub gene identification **(A)** Bar plots indicating the top 15 genes ranked by feature importance across four machine learning models (RF, XGB, GLM, SVM). **(B)** Comparison of cumulative residual curves across different predictive models. **(C)** Boxplots of residuals with red dots indicating root mean squared error (RMSE) for each model. **(D)** Receiver operating characteristic (ROC) curves illustrating the predictive accuracy of the four machine learning algorithms on the test dataset. **(E)** Venn diagram illustrating the intersection of important feature genes across the four models. **(F)** ROC curve analysis of candidate genes in the testing dataset.

To further screen for subtype-specific genes with strong diagnostic potential, we developed and validated four machine learning algorithms—random forest (RF), support vector machine (SVM), generalized linear model (GLM), and extreme gradient boosting (XGB)—using the expression profiles of 40 overlapping genes from the training dataset. Model interpretability was assessed using the DALEX package, and residual distribution plots were generated on the testing cohort for each algorithm. The top 15 predictive features for each model were ranked based on their root mean square error (RMSE) values (Figure 3A). Among the four models, SVM, GLM, and XGB demonstrated relatively lower residuals (Figures 3B,C). Five-fold cross-validation was subsequently performed to validate the model results, and ROC curve analysis was used to evaluate the discriminatory performance of the four algorithms on the test dataset. The AUC values for all models were comparably high (GLM, AUC = 0.997; SVM, AUC = 1; RF, AUC = 1; XGB, AUC = 1; Figure 3D). We then identified the intersection of the top 15 ranked features across all four models and selected four hub genes—G0S2, GZMA, ITM2A, and PAG1—for further analysis (Figure 3E). Model performance was further validated by applying five-fold cross-validation on the identified key genes. Among the four candidate genes, PAG1 exhibited the highest AUC value (AUC = 0.963), as shown in Figure 3F.

### 3.4 Expression profiles of hub genes

**FIGURE 4:**
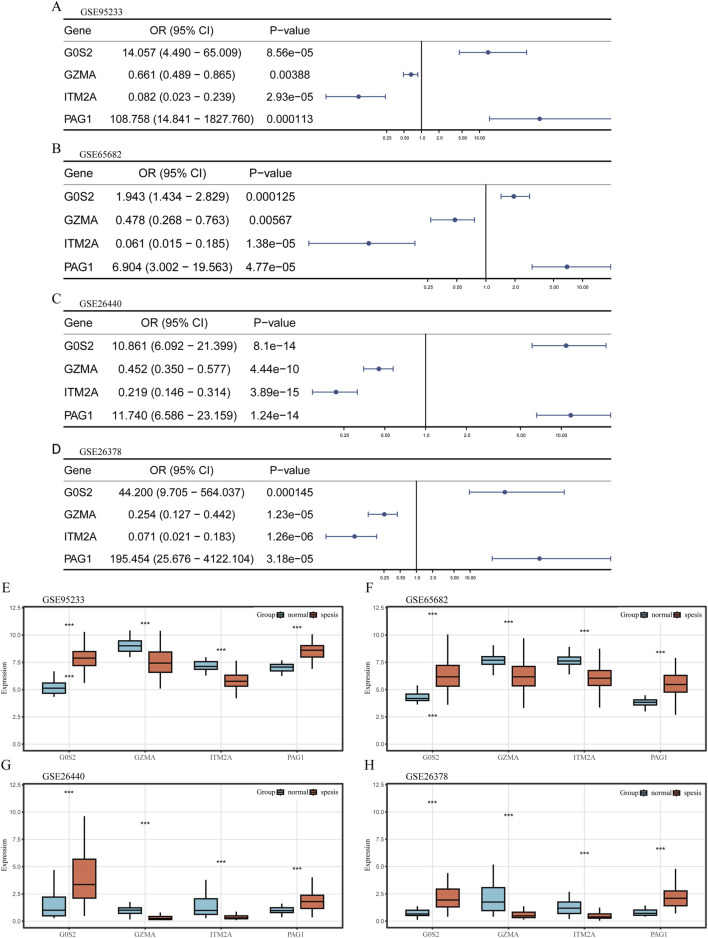
Expression profiling of hub genes **(A–D)** Univariate logistic regression forest plots depicting hub genes across different datasets. **(E–H)** Box plots illustrating the differential expression of hub genes between sepsis and control samples across various datasets. The plots display the median, interquartile range, and data distribution for each group.

Logistic regression analysis revealed that G0S2 and PAG1 functioned as risk factors, while GZMA and ITM2A served as protective factors across the GSE65682, GSE26440, GSE95233, and GSE26378 datasets (Figures 4A–D).

Transcriptomic profiling further demonstrated that G0S2 and PAG1 were highly expressed in sepsis samples, whereas GZMA and ITM2A were significantly downregulated in the same samples across all four datasets (Figures 4E–H).

**FIGURE 5:**
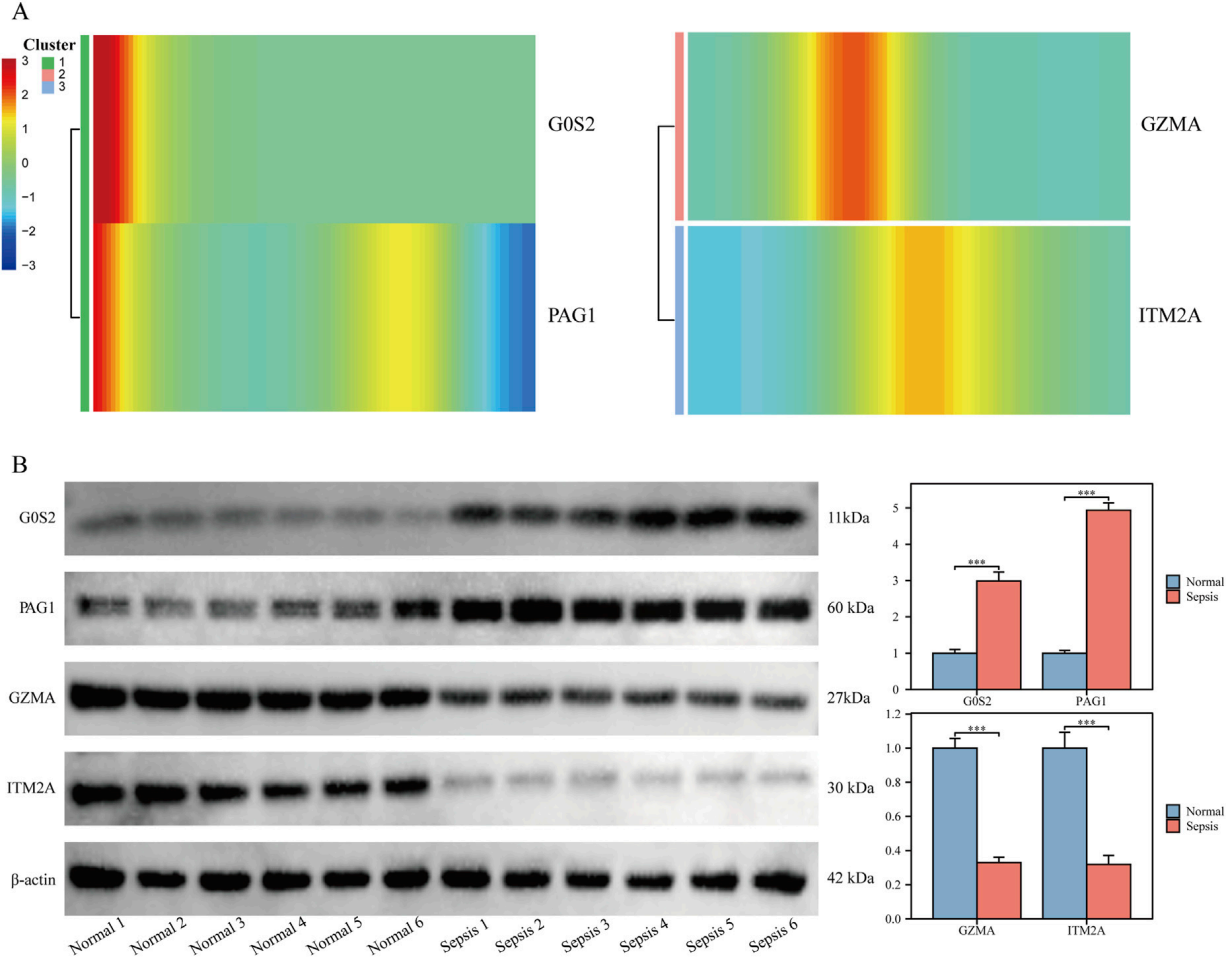
Validation of hub gene expression in clinical samples **(A)** Heatmap showing temporal dynamics of hub gene expression along pseudotime. **(B)** Western blot validation of hub gene protein expression in clinical samples.

Pseudotime trajectory analysis of hub genes showed that G0S2 and PAG1 were predominantly expressed at early pseudotime points, followed by a rapid decline, suggesting their potential roles in early inflammatory sensing and negative feedback regulation. GZMA exhibited a transient expression peak during the intermediate stage, which quickly diminished, indicating a potential short-lived intracellular or extracellular antimicrobial/apoptotic activation function. In contrast, ITM2A maintained elevated expression levels throughout the middle-to-late stages, possibly contributing to monocyte development and survival, and may play a role in counteracting prolonged inflammation (Figure 5A).

In clinical peripheral blood samples, protein levels of G0S2 and PAG1 were significantly upregulated, while GZMA and ITM2A were downregulated in monocytes isolated from sepsis patients, compared with healthy controls (P < 0.05 for all; Figure 5B).

### 3.5 Construction and validation of a predictive model for sepsis risk

**FIGURE 6:**
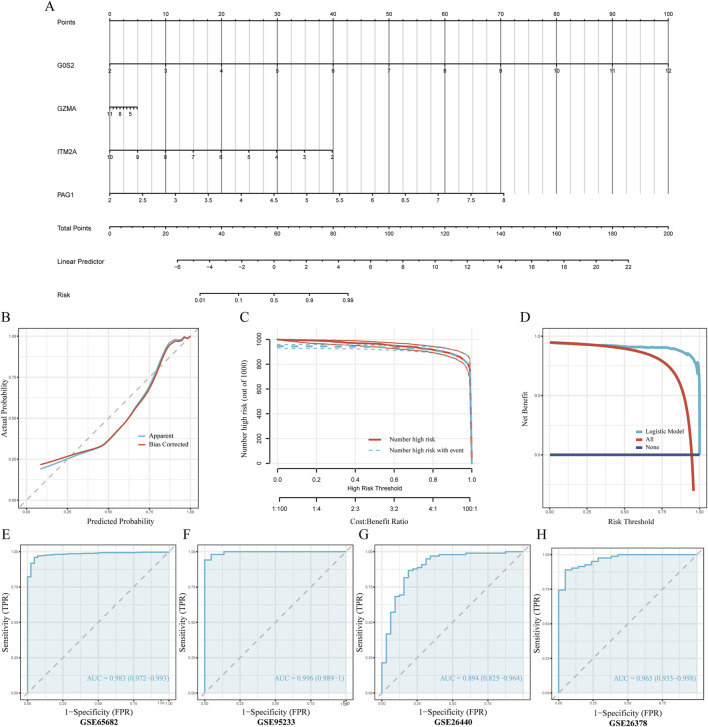
Development and validation of the nomogram-based predictive model **(A)** Nomogram diagnostic prediction model constructed using G0S2, ITM2A, PAG1, and GZMA. **(B)** Calibration curves assessing consistency between predicted and actual observed values. **(C)** Decision Curve Analysis (DCA) evaluating the clinical net benefit of the nomogram. The x-axis represents the threshold probability for diagnosing sepsis, while the y-axis represents the net benefit. The curve for our model (red line) shows a greater net benefit across a wide range of clinically relevant thresholds compared to the strategies of treating all patients (dashed gray line) or no patients (solid blue line), indicating its potential for clinical utility. **(D)** Clinical impact curve illustrating the practical application of the nomogram. At any given risk threshold on the x-axis, the red curve shows the number of individuals predicted to be high-risk, while the blue curve shows the number of true positive cases within that group. The close approximation of the two curves demonstrates the model’s strong performance in accurately identifying patients at high risk of sepsis in a clinical setting. **(E–H)** ROC curve analyses evaluating predictive accuracy of the model in the training and multiple validation datasets.

To predict sepsis susceptibility, we established a nomogram integrating G0S2, GZMA, ITM2A, and PAG1 expression levels (Figure 6A). Risk scores were computed according to the formula below: RiskScore = (G0S2 × 1.73) + (GZMA × −0.11) + (ITM2A × −0.86) + (PAG1 × 2.04). The calibration plot showed a strong concordance between predicted and actual sepsis probabilities, indicating excellent calibration performance of the model (Figure 6B). DCA indicated that, within the threshold probability range of 0.4–0.8, the model yielded a higher net clinical benefit than the strategies of treating all or none of the patients (Figure 6C). In addition, the clinical impact curve demonstrated that, over a high-risk threshold interval of 0.2–1.0, the number of individuals predicted as high-risk closely paralleled the number of actual positive cases, suggesting strong clinical applicability of the model (Figure 6D). Finally, ROC curves were plotted for the training cohort (GSE65682) and the validation cohorts (GSE26440, GSE95233, and GSE26378) to evaluate the predictive accuracy of the model. The results showed that all datasets achieved an AUC greater than 0.7, indicating good discriminative ability and predictive performance of the model (Figures 6E–H).

### 3.6 Immune infiltration analysis

**FIGURE 7:**
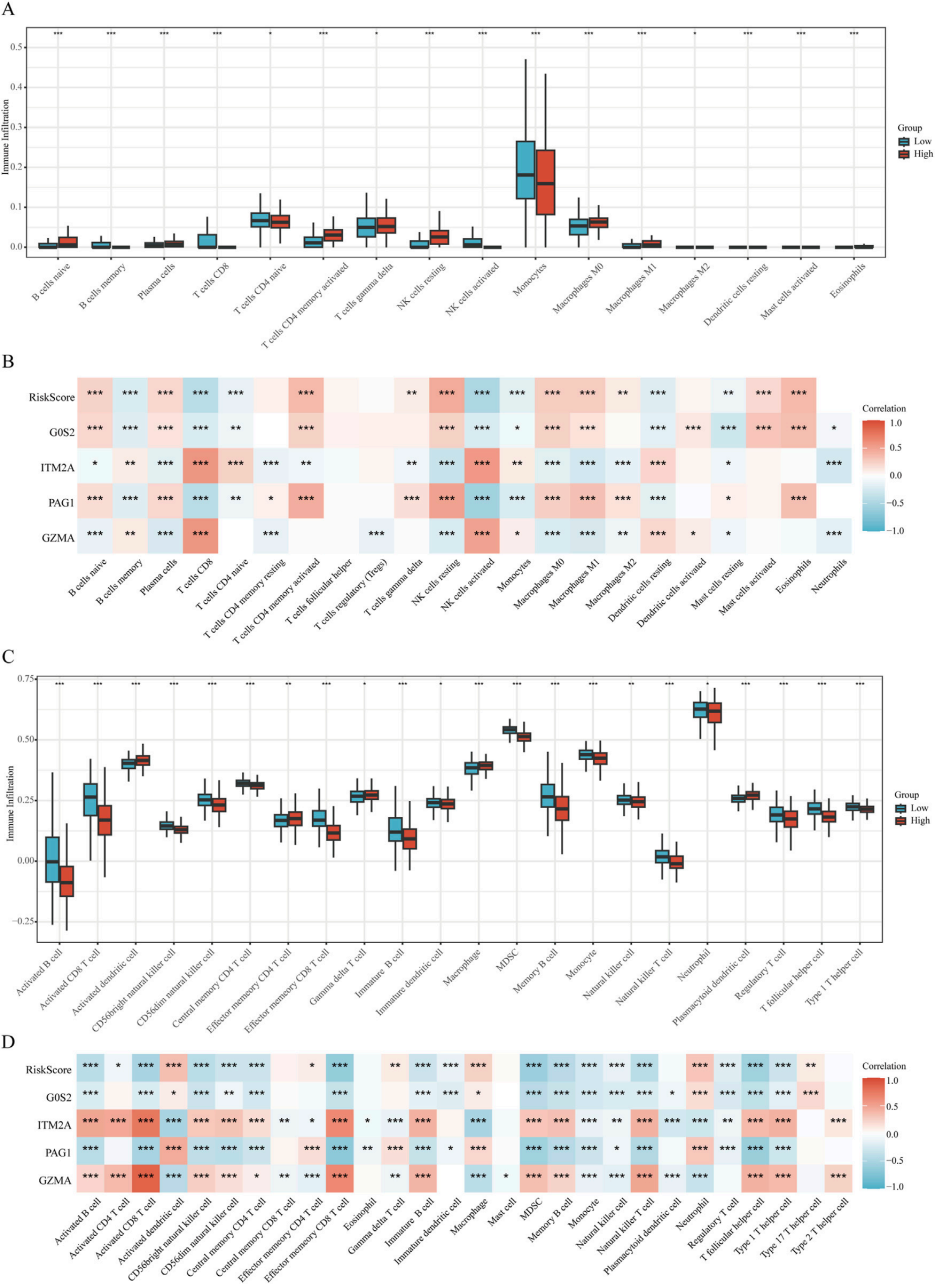
Immune microenvironment analysis **(A,B)** Correlation plots illustrating immune cell infiltration differences analyzed by CIBERSORT relative to the RiskScore. **(C,D)** Correlation plots illustrating immune cell infiltration differences analyzed by ssGSEA relative to the RiskScore.

The CIBERSORT algorithm revealed notable differences in immune cell infiltration between the risk groups. In the low-risk cohort, higher proportions of memory B cells, CD8^+^ T lymphocytes, naïve CD4^+^ T cells, activated natural killer (NK) cells, and monocytes were observed relative to the high-risk group. Conversely, the high-risk group displayed enhanced infiltration of naïve B cells, plasma cells, activated memory CD4^+^ T cells, γδ T cells, resting NK cells, as well as both M0 and M1 macrophage populations, along with eosinophils (Figures 7A,B).

Complementary results from ssGSEA indicated that individuals in the high-risk group exhibited significantly elevated enrichment scores for activated dendritic cells, effector memory CD4^+^ T cells, γδ T cells, macrophages, and plasmacytoid dendritic cells. In contrast, the low-risk group demonstrated greater abundance of diverse immune cell subsets, including activated B cells, activated CD8^+^ T cells, CD56^bright^ and CD56^dim^ NK cells, central memory CD4^+^ T cells, effector memory CD8^+^ T cells, immature B cells and dendritic cells, myeloid-derived suppressor cells (MDSCs), memory B cells, monocytes, NK cells, natural killer T (NKT) cells, neutrophils, regulatory T (Treg) cells, T follicular helper (Tfh) cells, and Th1 cells (Figures 7C,D).

### 3.7 GSEA analyse

**FIGURE 8:**
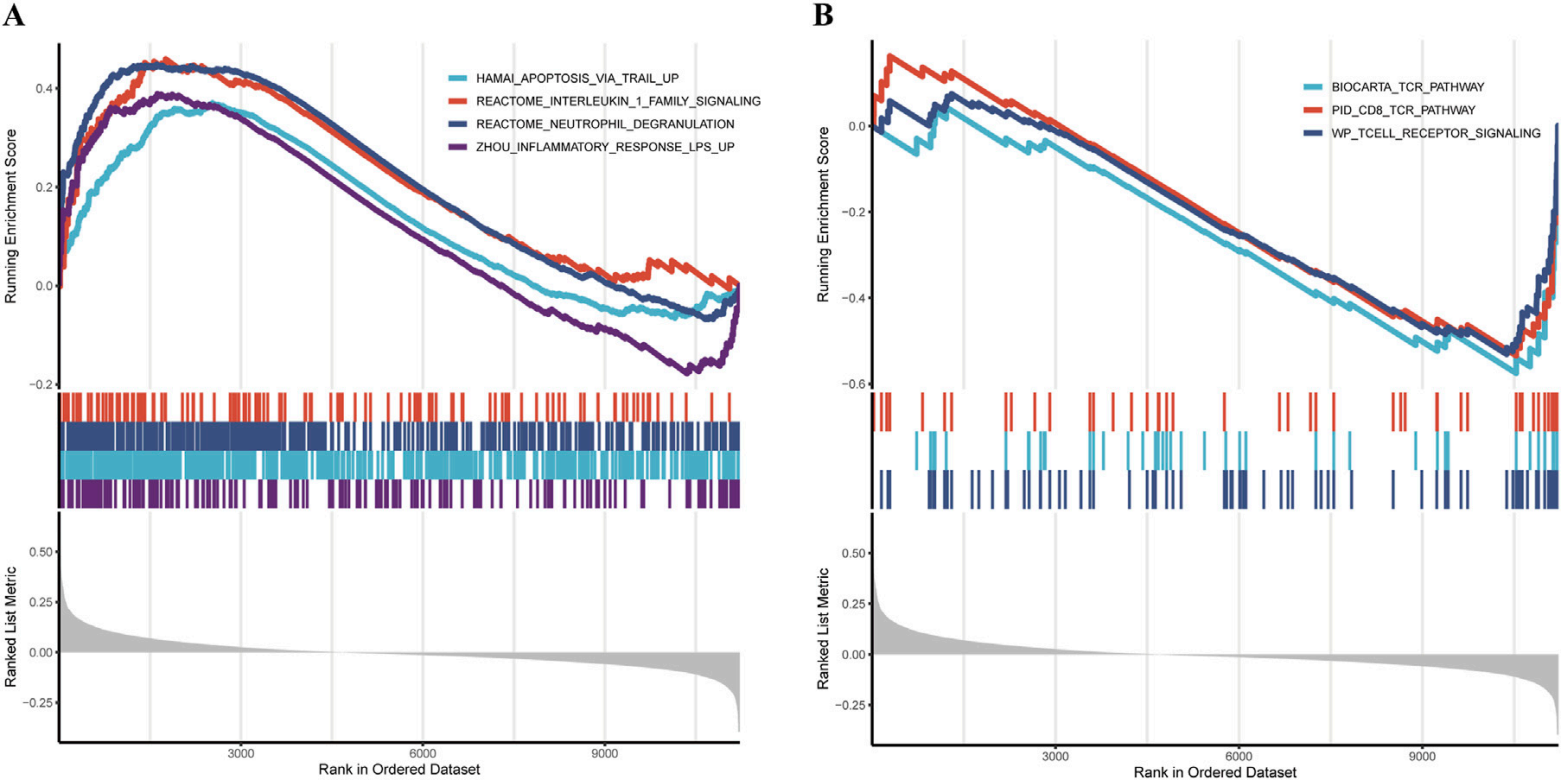
Pathway and functional analysis **(A,B)** GSEA revealing distinct pathway enrichment between high- and low-risk groups.

GSEA analysis revealed that the high-risk group was significantly enriched in pathways associated with inflammation and immune dysregulation, including:

REACTOME_NEUTROPHIL_DEGRANULATION, ZHOU_INFLAMMATORY_RESPONSE_LPS_UP, REACTOME_INTERLEUKIN_1_FAMILY_SIGNALING, In addition, the apoptosis-related pathway HAMAI_APOPTOSIS_VIA_TRAIL_UP was also markedly enriched in the high-risk group (Figure 8A).

Conversely, the low-riskscore group showed significant enrichment of pathways related to cellular homeostasis and immune regulation, such as: WP_TCELL_RECEPTOR_SIGNALING, BIOCARTA_TCR_PATHWAY, PID_CD8_TCR_PATHWAY (Figure 8B).

## 4 Discussion

Sepsis is a critical clinical syndrome characterized by organ dysfunction due to an imbalanced immune response to infection, often leading to high mortality (Singer et al., 2016). It poses a significant burden on global health systems and imposes a substantial burden on healthcare systems. Studies have shown that the incidence and mortality of sepsis are particularly high in developing and under-resourced regions (Adhikari et al., 2010). Although clinical interventions such as antimicrobial therapy, fluid resuscitation, and organ support have been widely implemented, the mortality associated with severe sepsis and septic shock remains unacceptably high. Therefore, early identification of sepsis is critical for reducing mortality and improving patient outcomes.

This study first identified 12 core immune cell populations based on scRNA-seq data, among which naïve CD4^+^T cells, natural killer cells, and classical monocytes were notably enriched in the context of sepsis. Pseudotime trajectory analysis revealed that classical monocytes were positioned at the main developmental branch, spanning the early, middle, and late stages of sepsis progression, suggesting their pivotal role in the disease course. As key effectors of innate immunity (Tahir and Steffens, 2021), classical monocytes are central to the early inflammatory response, mediating pathogen recognition, chemotaxis, phagocytosis, and antigen presentation, and serve as important nodes within the inflammatory signaling network (Jakubzick et al., 2017). Single-cell communication network analysis indicated that classical monocytes exhibited a “low-sender, high-receiver” signaling profile. This suggests that classical monocytes are not conventional initiators of inflammation but rather function as “responders” influenced by the surrounding inflammatory microenvironment. Their apoptotic process appears to be not solely driven by intrinsic factors but is regulated by external signaling pathways, such as GALECTIN and THBS, which are involved in apoptosis, adhesion, and chemotaxis. This may help explain the impaired functional capacity of classical monocytes in sepsis and provides critical cellular and molecular evidence for understanding the state of “immunoparalysis” (Martin et al., 2020) observed in septic patients.

Subsequently, a four-gene diagnostic model comprising G0S2, GZMA, ITM2A, and PAG1 was established through ROC regression analysis and four machine learning algorithms. The model demonstrated excellent calibration performance, with high concordance between predicted and observed sepsis risk. Unlike previous models (Liu et al., 2023), a key feature of our approach was the integration of results from multiple datasets and algorithms, followed by validation using both internal and external cohorts that were independent of the training set. The model achieved AUC values ranging from 0.997 to 1.000, indicating high reliability and robustness. Experimental validation further confirmed that G0S2 and PAG1 were significantly upregulated in sepsis and functioned as risk factors, whereas GZMA and ITM2A were downregulated and served as protective factors. G0S2 was initially identified in blood monocytes involved in cell cycle progression and is now recognized for its dual roles in inflammation and apoptosis, as well as its critical regulatory function in lipid metabolism (Heckmann et al., 2013). According to Kobayashi et al., G0S2 expression was significantly elevated in PBMCs obtained from individuals with systemic autoimmune vasculitis (Kobayashi et al., 2008). PAG1 is a transmembrane adaptor protein primarily known for its role in negatively regulating Src family kinases (SFKs) (Foltz et al., 2020; Koutros et al., 2023; Sun et al., 2023). The activation of TCR and BCR signaling pathways in T and B lymphocytes relies heavily on the precise regulation of Src family kinases (SFKs) (Lu et al., 2021). Therefore, alterations in PAG1 expression or function are likely to influence monocyte responses under inflammatory conditions such as sepsis.

Conversely, GZMA and ITM2A were downregulated in sepsis and identified as protective factors. GZMA is a serine protease secreted by T cells and NK cells and is considered a key effector molecule involved in the cytolytic activity of cytotoxic T lymphocytes and NK cells. Studies have shown that GZMA plays an important role in regulating the inflammatory response during peritoneal sepsis (Garzón-Tituaña et al., 2021). Inhibition of GZMA expression has been reported to significantly attenuate *Escherichia* coli–induced inflammatory responses and improve survival in animal models (Uranga-Murillo et al., 2021). Our finding that GZMA acts as a protective factor, as indicated by its downregulation in sepsis patients and negative correlation with risk score, may seem counterintuitive given its pro-inflammatory roles in other contexts. This discrepancy could be explained by the dual nature of granzymes and the specific cellular context of our study (Garzón-Tituaña et al., 2020). While high extracellular GZMA can drive inflammation and cell death, its primary function within cytotoxic lymphocytes is crucial for controlled pathogen clearance. In the context of sepsis-induced immunoparalysis, the observed downregulation of GZMA in monocytes may reflect a broader suppression of cytotoxic cell functions, leading to impaired infection control and thus a poorer prognosis. Therefore, in this setting, lower GZMA expression may not be directly protective but rather a marker of a dysfunctional immune state, making its relative abundance a “protective” indicator in our risk model. This highlights the context-dependent role of GZMA in sepsis. ITM2A is a type II transmembrane protein composed of 263 amino acids and shares high homology with ITM2B and ITM2C (Rengaraj et al., 2007). It is involved in various biological processes, including autophagy flux regulation, cartilage differentiation, and lipid metabolism (Van den Plas and Merregaert, 2004; Namkoong et al., 2015; Davies et al., 2017). In the immune system, ITM2A expression is closely associated with the function of multiple immune cell types. Downregulation of ITM2A has been observed in macrophages from patients with ankylosing spondylitis, suggesting a potential role in disease pathogenesis and inflammatory regulation (Lari et al., 2021).

Based on the sepsis diagnostic prediction model, this study successfully identified distinct immune characteristics associated with different risk groups, further emphasizing the close relationship between apoptosis and immune function. The results of CIBERSORT and ssGSEA analyses revealed that the low-riskscore group was characterized by significant enrichment of immune effector cells, such as CD8^+^T cells, monocytes, and activated NK cells, suggesting that enhanced pathogen clearance and efficient removal of apoptotic cells may contribute to improved clinical outcomes in sepsis. Previous studies have demonstrated that T cells—particularly CD8^+^ cytotoxic T lymphocytes—play a critical role in pathogen clearance by secreting effector molecules including perforin, interferon-gamma (IFN-γ), and granzymes, thereby effectively eliminating infectious agents from the host (Diao et al., 2020). Conversely, individuals in the high-risk group showed a significant elevation in the proportion of M0 and M1 macrophages, indicating that excessive inflammatory activation combined with dysregulated apoptotic control may contribute to exacerbated tissue damage and disease progression in sepsis. It is well established that M1 macrophages are central mediators of pro-inflammatory responses and immune activation (Kadomoto et al., 2021). During the acute inflammatory phase of early sepsis, targeting the overactivation of M1 macrophages has been shown to effectively reduce the release of inflammatory cytokines, thereby mitigating tissue injury and lowering the risk of mortality (Wang and Wang, 2023).

GSEA analysis further elucidated the key biological pathways underlying risk stratification. In the high-risk group, pronounced inflammatory activity was suggested by the enrichment of the Reactome neutrophil degranulation pathway and the Zhou LPS-induced inflammatory response gene set, which may contribute to widespread cellular injury and tissue destruction, thereby accelerating disease progression. Previous studies have demonstrated that excessive neutrophil degranulation leads to the release of large quantities of pro-inflammatory mediators, markedly exacerbating immune dysregulation and contributing to multi-organ dysfunction in sepsis (Zhang et al., 2023). Moreover, the enrichment of the Hamai apoptosis via TRAIL upregulated pathway suggested an elevated level of apoptotic activity within the high-risk group. Conversely, the low-risk group showed significant enrichment in several T cell receptor–related signaling pathways, including the WP T cell receptor signaling, BioCarta TCR pathway, and PID CD8 TCR pathway, reflecting more active and functional T cell responses. This may enhance immune surveillance and antimicrobial defense, thereby restraining the spread of inflammation and preventing tissue damage. These findings are consistent with previous reports showing that the preservation of T cell functionality is closely associated with improved clinical outcomes in patients with sepsis (Heidarian et al., 2023; Sossou et al., 2024).

Crucially, this stratification holds direct therapeutic implications. For instance, high-risk patients, characterized by hyperinflammation and excessive apoptosis (e.g., elevated neutrophil degranulation and TRAIL-pathway activity), may benefit from targeted anti-inflammatory agents or apoptosis inhibitors (Zeng et al., 2022). Conversely, low-risk patients with robust T-cell responses might be candidates for therapies that preserve or enhance adaptive immunity, avoiding broad immunosuppression. Our four-gene signature thus provides a molecular rationale for tailoring immunomodulatory interventions, moving beyond a one-size-fits-all approach toward true precision medicine in sepsis.

This study achieved preliminary progress in constructing a diagnostic model for sepsis and identifying key genes; however, several limitations remain. First, the data were derived from retrospective datasets obtained from public databases, which may introduce selection bias and heterogeneity. In addition, the clinical samples used for Western blot validation were limited in number (n = 6 per group), which serves as a preliminary verification and may restrict the generalizability of the findings. To robustly establish the clinical utility and prognostic value of our four-gene signature, further validation in large-scale, prospective multicenter cohorts is necessary. Such studies should also incorporate key clinical outcomes, including 28-day mortality and organ dysfunction scores, to fully evaluate the model’s potential to guide patient management. Second, although we validated the differential expression of the four hub genes at the protein level, the study did not include functional experiments to directly probe their mechanistic roles. The specific molecular pathways by which these genes regulate apoptosis in classical monocytes during sepsis—including their upstream regulators and downstream effectors—remain to be elucidated. Future *in vitro* studies, such as siRNA-mediated knockdown or CRISPR-based modulation of these genes in monocyte cell lines, coupled with functional assays for apoptosis and inflammatory cytokine production, are warranted. These experiments would be crucial for confirming a causal relationship and dissecting the underlying regulatory networks. Moreover, this study primarily focused on diagnostic value, without evaluating the predictive capacity of the model with respect to clinical outcomes such as mortality or organ dysfunction. The analysis was restricted to classical monocytes, and the roles of these genes in other immune or non-immune cell types were not explored, indicating a limitation in cellular scope. Finally, the functional validation remains at the level of literature review and GSEA-based inference, lacking direct experimental evidence to support the biological roles of these genes in sepsis.

In conclusion, this study combined single-cell and bulk RNA sequencing data with multiple machine learning approaches to uncover four key hub genes—G0S2, GZMA, ITM2A, and PAG1—that are closely associated with apoptosis in classical monocytes during sepsis. The diagnostic model constructed based on these four genes demonstrated robust discriminative performance and high diagnostic accuracy in both the training set and multiple independent validation cohorts. This work not only provides a promising panel of biomarkers for the early diagnosis of sepsis but also offers new research directions and theoretical insights into the pivotal role of classical monocytes in the pathogenesis of sepsis.

## Data Availability

Publicly available datasets were analyzed in this study. This data can be found here: The datasets analyzed in this study are publicly available in the Gene Expression Omnibus (GEO) repository: GSE65682: https://www.ncbi.nlm.nih.gov/geo/query/acc.cgi?acc&equals; GSE65682 GSE26440: https://www.ncbi.nlm.nih.gov/geo/query/acc.cgi?acc&equals; GSE26440 GSE95233: https://www.ncbi.nlm.nih.gov/geo/query/acc.cgi?acc&equals; GSE95233 GSE26378: https://www.ncbi.nlm.nih.gov/geo/query/acc.cgi?acc&equals; GSE26378 GSE167363: https://www.ncbi.nlm.nih.gov/geo/query/acc.cgi?acc&equals; GSE167363. The complete source code and data analysis pipeline are openly available on GitHub: https://github.com/Duanwj112/Duanwj_sepsis.
