# Supplementary figures and images for: Decoding monocyte heterogeneity in sepsis: a single-cell apoptotic signature for immune stratification and guiding precision therapy
Source: Front Pharmacol. 2025 Oct 3;16:1675887. doi: 10.3389/fphar.2025.1675887 (PMC12531134; doi:10.3389/fphar.2025.1675887)

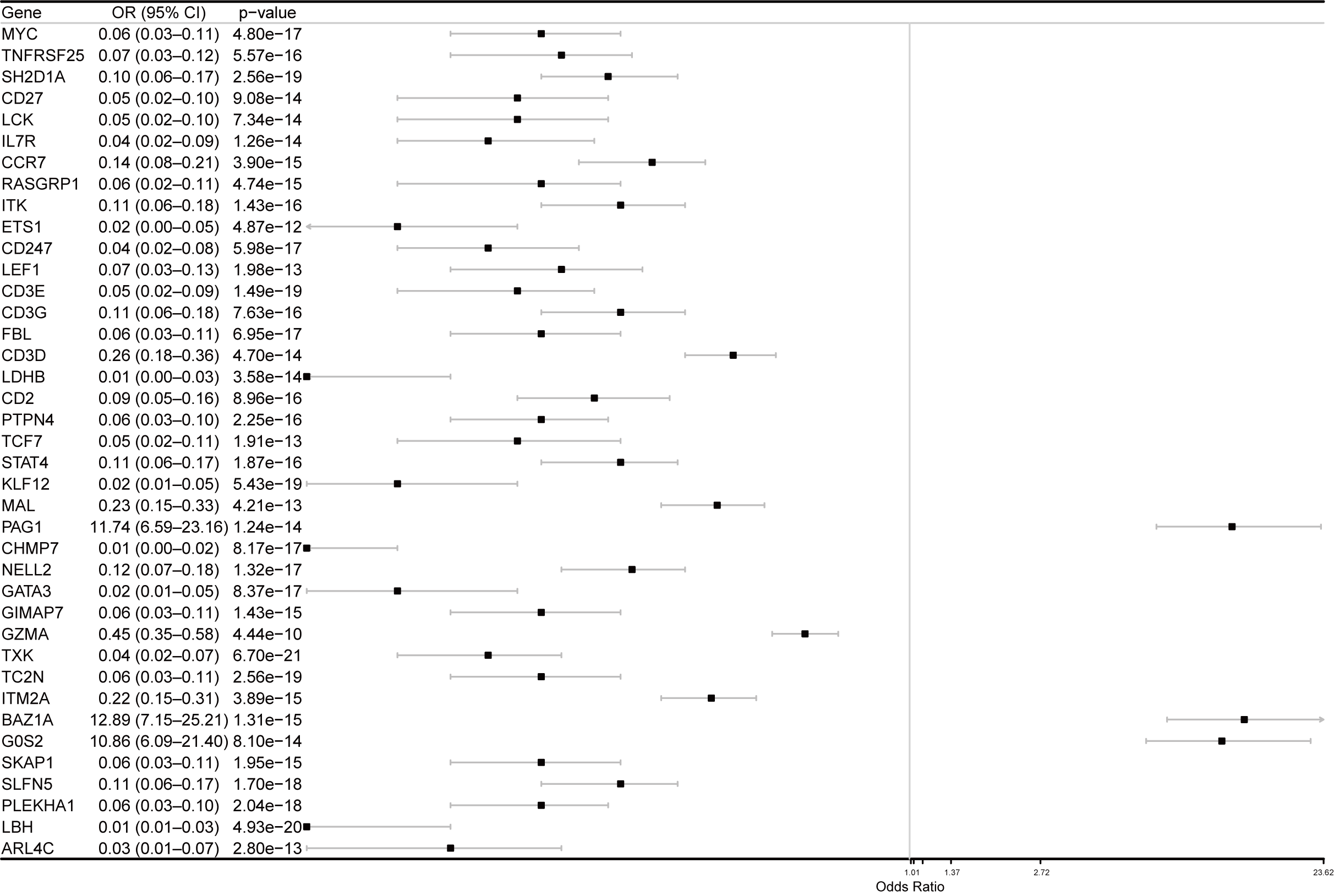

Supplement: Supplementary file 1 [file Image1.tif]
